# Editorial: Plant Disease Management in the Post-genomic Era: From Functional Genomics to Genome Editing

**DOI:** 10.3389/fmicb.2020.00107

**Published:** 2020-02-04

**Authors:** Sabrina Sarrocco, Alfredo Herrera-Estrella, David B. Collinge

**Affiliations:** ^1^Department of Agriculture, Food and Environment, University of Pisa, Pisa, Italy; ^2^National Laboratory of Genomics for Biodiversity, Center for Research and Advanced Studies of the National Polytechnic Institute, Irapuato, Mexico; ^3^Section for Microbial Ecology and Biotechnology, Department of Plant and Environmental Sciences and Copenhagen Plant Science Centre, University of Copenhagen, Copenhagen, Denmark

**Keywords:** disease management, NGS - next generation sequencing, food security, food safety, post-genomic era

The growing world population requires an efficient management and control of diseases in crop production to guarantee both food security and safety (FAO, 2018; Sarrocco and Vannacci, 2018). The development of the so called NGS (Next Generation Sequencing) techniques has been positively welcomed as a new tool for understanding the nature of plant diseases, even if the potential for their use has not yet been fully discovered.

This Research Topic arises from the idea to give an updated and exhaustive overview of the exploitation of genome sequencing, genome comparison, transcriptomics, metagenomics, RNA based technologies, and genome editing strategies as a new frontier to contribute to plant disease management.

The knowledge of the complex relationship occurring among plants, pathogens, the environment and, eventually, beneficial organisms, is now increasing thanks to the ecological application of genomics (ecogenomics), both at single strain and at community level (Martin, 2014). Metagenomics and metatranscriptomics can be of help to describe the whole microbial community not only in terms of its ecology, but also to detect that fraction of the microbiome that, modulating the activity of plant pathogens in favor of the plant host, could be developed (as single isolate or in consortia) as biopesticides. Cobo-Díaz et al. used a concurrence culture-independent metabarcoding approach to characterize the microbial communities associated to the incidence and composition of *Fusarium* spp. on maize stalk and other bacterial and fungal genera, using co-occurrence network analysis. Such approach could be a useful tool as part of a screening strategy for novel antagonist candidates against toxigenic *Fusarium* spp.

The wide range of beneficial microorganisms does not only include those isolates directly interacting with harmful pathogens but also consists of endophytes that, thanks to their mutualistic relationship with the host, can increase plant tolerance to biotic and abiotic stress. Pereira et al. grouped into operational taxonomic units (OTU) ITS sequences amplified from culturable fungi associated to *Festuca rubra* subsp. *pruinosa* (FRP) roots thus demonstrating a set of seven species. These seem to be the components of the core mycobiome of FRP and include very promising candidates in the adaptation of FRP plants to salinity, a characteristic stress factor of their habitat. Indeed a *Diaporthe* strain could help ryegrass (*Lolium*) to adapt to high salinity. The genome of bacterial endophyte *Paraburkholderia phytofirmans* was extensively studied by Esmaeel et al., allowing the identification of all gene clusters which contribute to the adaptive mechanisms under different environmental conditions and explaining the high ecological competence of this microorganism, able to promote plant growth and to induce resistance to abiotic and biotic stresses.

The number of fully-sequenced and released genomes is increasing rapidly. This genomics “revolution” gave an important contribution in plant pathology, rapidly increasing our knowledge of the molecular mechanisms underpinning pathogenesis, resistance and the mode of action of beneficial microorganisms (Klosterman et al., 2016). Firrao et al., through the analysis of Illumina sequence data-sets of 11 European and one non-European *Peudomonas syringae* pv. *actinidia* (Psa) genomes, gave a picture of the significant differences in the genome evolution of this bacterium before and after a clonal expansion, thus furnishing information of great value for epidemic management. In the same way, the genome-wide analysis of the plant pathogenic bacteria *Ralstonia solanacearum* and *Xantomonas oryzae* pv. *oryzae*, performed by Cho et al., and Doucouré et al., respectively, will have important effect in the sustainable deployment of broad-spectrum and durable resistance to these serious pathogens.

Broberg et al., by performing the re-sequencing of the genome of 52 isolates of the beneficial fungus *Clonostachys rosea*, made a genome-wide association study in conditions relevant for biocontrol activity, with particular attention to the cold tolerance of these fungi, an important implication for the management of plant diseases.

Also from the host-plant side, genomics and post-genomics techniques are actually giving wide opportunities to understand diseases and stress tolerance, as well as to design innovative control strategies, as overviewed by Leonetti et al. on chickpea. The contribution of plant proteomics is no less important for understanding mechanisms underlining resistance to common diseases, such as in the pathosystem *Fusarium oxysporum*/tomato, where defense gene response was phenotypically characterized by de Lamo et al..

During plant/microorganism interactions, plant colonization is governed by secreted effectors that can belong to the same families in the case both of harmful (phytopathogens) and beneficial (symbiontic) interactions. The role of effectors in the molecular chat occurring with *Trichoderma* isolates, as well as during arbuscular mycorrhizal (AM) symbiosis—that facilitates mineral nutrition and confers tolerance to biotic and abiotic stresses—was discussed by Ramirez-Valdespino et al. and Voß et al., respectively.

Progresses in genetic engineering created new opportunities in plant disease management. This is the case of RNA interference (RNAi) as well as genome editing technologies that, with a rapid progress, has become an important genetic tool to “*touch up*” the genome of host plants or pathogens, as well as of beneficial microorganisms (Collinge, 2018; Nødvig et al., 2018).

In filamentous fungi, as example, gene silencing mediated by RNAi, models important biological processes, including pathogenicity. Having previously demonstrated that it works (Koch et al., 2013). Gaffar et al. explored RNAi machinery in *Fusarium graminearum* for sexual-asexual reproduction, sensitivity to double-strand (ds)RNA and pathogenicity in order to develop RNAi-based plant protection strategies.

The CRISPR/Cas9 technology applied to plants was reviewed by Borrelli et al. as a tool to implement pathogen resistance, thus representing the natural consequence of years spent in deciphering and reading genomes. The possibility to edit and re-write fungal genomes described by Vicente Muñoz et al., is an additional possibility of controlling plant pathogens by creating new interesting biocontrol strains to be released in field thus avoiding the introduction of transgenes in the environment.

The availability of NGS and all the developing “omics” offer new tools for an environmentally and economically sustainable crop protection by improving our knowledge of the complex network, at cell, individual and ecosystem level, modulating plant diseases and by offering us new tools to rapidly react to new, emerging and re-emerging plant diseases in a fast changing environment.

## Author Contributions

All authors listed have made a substantial, direct and intellectual contribution to the work, and approved it for publication.

### Conflict of Interest

The authors declare that the research was conducted in the absence of any commercial or financial relationships that could be construed as a potential conflict of interest.
